# Post-pituitary surgery copeptin analysis as a ‘rule-out’ test for post-operative diabetes insipidus

**DOI:** 10.1007/s12020-022-03220-7

**Published:** 2022-10-22

**Authors:** Hussam Rostom, Sean Noronha, Bahram Jafar-Mohammadi, Christine May, Anouk Borg, Jane Halliday, Simon Cudlip, Tim James, Nishan Guha, Brian Shine, Aparna Pal

**Affiliations:** 1 Department of Clinical Biochemistry, Oxford University Hospitals NHS Foundation Trust, Headley Way, Oxford, OX3 9DU UK; 2 Oxford Centre for Diabetes, Endocrinology and Metabolism, Churchill Hospital, Old Road, Headington, Oxford, OX3 7LE UK; 3 Department of Neurosurgery, Oxford University Hospitals NHS Foundation Trust, Headley Way, Oxford, OX3 9DU UK

**Keywords:** Copeptin, AVP, Diabetes insipidus, Pituitary, Neurosurgery

## Abstract

**Background:**

Diabetes insipidus (DI) is a recognised complication of pituitary surgery, with diagnosis requiring clinical observation aided by plasma and urine electrolytes and osmolalities. Copeptin is a stable surrogate marker of AVP release and has potential to facilitate prompt diagnosis of post-operative DI. This assay has been shown to accurately predict which patients are likely to develop DI following pituitary surgery.

**Objective:**

To determine whether copeptin analysis can be used to predict which patients are at risk of developing DI following trans-sphenoidal surgery (TSS).

**Methods:**

Seventy-eight patients undergoing TSS had samples taken for copeptin pre-operatively and at day 1 post-TSS. The majority of patients also had samples from day 2, day 8, and week 6 post-TSS. Results from patients who developed post-operative DI (based on clinical assessment, urine and plasma biochemistry and the need for treatment with DDAVP) were compared to those who did not. Patients with any evidence of pre-operative DI were excluded.

**Results:**

Of 78 patients assessed, 11 were clinically determined to have developed DI. Differences were observed between patients with DI and those without in post-operative samples. Of note, there was a significant difference in plasma copeptin at day 1 post-operation (*p* = 0.010 on Kruskal–Wallis test), with copeptin levels greater than 3.4 pmol/l helping to rule out DI (91% sensitivity, 55% specificity at this cut off).

**Conclusion:**

In the post-TSS setting, copeptin is a useful rule-out test in patients with values above a defined threshold, which may facilitate earlier decision making and shorter hospital stays.

## Introduction

Central diabetes insipidus (DI) following pituitary surgery is a well-recognised complication, often occurring early and transiently post-operatively with incidence variably documented as ranging from 1 to 67% [1]. Such post-operative DI is associated with increased morbidity and a greater length of hospital stay [2] and accurate diagnosis is essential to allow early treatment. Currently this relies upon a combination of clinical assessment, fluid balance monitoring, and plasma and urine sampling for sodium and osmolality analysis [3].

Efficient diagnosis of DI is limited by the challenge of directly assaying arginine vasopressin (AVP), which has a short half-life in vivo [4], is unstable ex vivo even when frozen [5], and is affected by delayed or incomplete separation from platelets [6]. As a result, there are no AVP assays available in clinical laboratories in the United Kingdom. Copeptin is the 39 amino acid C-terminal segment of pre-pro-AVP, and is produced in isomolar quantities to AVP [7]. It can be measured using commercially available immunoassay methods, and has the major advantage of being much more stable than AVP in blood after venesection. Copeptin has been demonstrated to add diagnostic accuracy in the investigation of polyuria-polydipsia syndrome [8, 9], and Winzeler et al. showed that patients with DI after pituitary surgery had a lower copeptin level than those who did not develop DI [10].

The aims of our study were to determine the predictive value of copeptin in diagnosing DI after trans-sphenoidal surgery (TSS) in our centre, to assess its utility in routine clinical practice, and to explore using a discriminative ‘cut-off’ to facilitate earlier discharge.

## Methods

### Patient selection

Plasma samples were collected from 78 patients undergoing elective TSS at the John Radcliffe Hospital (Oxford University Hospitals NHS Foundation Trust, Oxford, UK) between November 2017 and June 2020. All patients had pituitary tumours or other lesions within the pituitary fossa. Patients were excluded if they had a diagnosis of pre-operative DI.

To determine the required sample size, we used existing data on copeptin values in the post-TSS setting [10], and established methods for estimating mean and SD from mean and IQR [11]. We obtained Cohen’s *d* effect size of 0.83, and used an *α* error of 0.05, a power of 0.8, and an allocation ratio of 6 (giving *n* = 76 patients required, with *n* = 11 with DI and *n* = 65 without DI).

Baseline characteristics, including gender, age, tumour type, lesion size and renal function were obtained from electronic patient records.

### Lesion characteristics

Patients underwent pre-operative magnetic resonance imaging of the pituitary with contrast and all scans were reported by a Neuroradiologist. Lesions <1 cm in diameter were classified as microadenomas and those ≥1 cm in diameter classified as macroadenomas. Following resection, tumour histology was determined and reported by Neuropathologists based on immunohistochemical characteristics.

### Surgery and post-operative protocol

All TSS were carried out by a single Neurosurgical team. Operations were carried out under general anaesthetic using endoscopic techniques.

Patients remained in hospital for a minimum of 48 hours, and were fluid restricted to 2 litres per day post-operatively, to prevent masking of biochemical abnormalities through compensatory drinking. During admission, blood and urine samples were obtained routinely at day 1 and day 2 post-operatively to assess for biochemical evidence of DI. If there was clinical suspicion of DI, urgent laboratory samples were sent and treatment promptly initiated, pending biochemical confirmation.

### Diagnosis and treatment of DI

DI was suspected if patients had a urine output of more than 200 ml/h for three consecutive hours or more than 3 litres per day, and this was confirmed with biochemistry showing high plasma sodium (>145 mmol/l) and osmolality (>295 mosmol/kg) with inappropriately low urine osmolality. If DI was suspected, fluid restriction was immediately lifted and urgent biochemistry samples sent. If biochemistry confirmed DI, 1 microgram of DDAVP was administered subcutaneously. If DI persisted more than 24 hours after DDAVP administration and biochemistry confirmed ongoing DI, patients were discharged on desmopressin. Patients were re-assessed at day 8 and 6 weeks after their operation. At follow up, desmopressin was stopped for 24 hours before assessment and patients allowed to drink to thirst. Patients who required ongoing desmopressin treatment beyond 3 months were classified as ‘permanent’ DI, while those who had no further need for desmopressin were classified as having ‘transient’ DI.

### Sample collection

Patients undergoing TSS have samples taken for routine biochemistry pre-operatively, and subsequently at day 1, day 2, day 8, and week 6 post-TSS. Day 1 samples were typically taken on the morning after surgery. Aliquots at each of these time points were frozen for retrospective copeptin batch analysis.

### Laboratory analysis

Plasma and urine sodium analysis was performed on the Abbott Architect c16000 (Abbott Diagnostics), which uses an indirect ion-selective electrode method. Osmolality was determined using the Model 3320 Osmometer (Advanced Instruments), which assesses osmolality by measuring freezing-point depression.

Copeptin was analysed using the Brahms KRYPTOR immunofluorescence assay (Thermo Fisher Scientific), with reproducibility of 6.8% coefficient of variation (CV) at 5.1 pmol/l and 3.9% CV at 99.3 pmol/l.

### Comparison

Patients were divided into groups depending on whether they developed post-operative DI (based on clinical assessment, urine and plasma biochemistry and the need for treatment with DDAVP), or not. Copeptin results pre-operatively and at day 1, day 2, day 8 and week 6 post-operatively were compared between groups to see if there was a significant difference.

### Statistical analysis

Since copeptin values were not normally distributed, we compared medians between DI and non-DI groups using the Kruskal–Wallis test. Data are presented graphically using box-whisker plots, and we obtained optimal cut-off levels using receiver-operator characteristic (ROC) curves. Comparison of copeptin values at different observation times in each group was performed using the Friedman test. We compared baseline characteristics using chi-squared analysis for categorical data and the Kruskal–Wallis test for age. Statistical analysis was performed using R software, version 4.0.0 (R Core Team (2020). R: A language and environment for statistical computing. R Foundation for Statistical Computing, Vienna, Austria. URL https://www.R-project.org/).

## Results

### Baseline characteristics

Baseline characteristics of the cohort are shown in Table 1. The 78 patients undergoing TSS had a median age of 55 (range 22–85), with 41 of 78 being men (52.6%). Two patients had estimated glomerular filtration rates <30 ml/min/1.73 m^2^, two had heart failure, and 11 had type 2 diabetes mellitus, but none of these developed DI. The majority of lesions (72/78; 92.3%) were macroadenomas, while histologically gonadotroph tumours were the most common (37/78; 47.4%).

**Table 1:** Baseline characteristics of participants

	No. of patients (*n* = 78)	Percentage
Gender		
Male	41	52.6%
Female	37	47.4%
Co-morbidities		
Severe chronic kidney disease (eGFR < 30)	2	2.6%
Diabetes mellitus	11	14.1%
Heart failure	2	2.6%
Histology		
Gonadotroph	37	47.4%
Corticotroph	6	7.7%
Somatotroph	7	9.0%
Lactotroph	4	5.1%
Plurihormonal	8	10.3%
Hormone inactive	2	2.6%
Craniopharyngioma	3	3.8%
Rathke’s cyst	6	7.7%
Other	5	6.4%
Lesion size ≥ 1 cm	72	92.3%

The gender, renal function, histology and lesion size of *n* = 78 participants included in the study are shown, as well as the percentage with each characteristic. ‘Other’ histological diagnoses were: non-neoplastic pituitary tissue (*n* = 2), meningothelial tumour, fibrotic tissue with lymphocytic infiltrate, foreign-body material (no native tissue)

### Incidence of diabetes insipidus

Of the 78 patients included, 11 (14.1%) were diagnosed with DI post-operatively and required treatment with desmopressin. Of these, seven (9.0%) remained on desmopressin treatment after 3 months and were presumed to have permanent DI, while the other four (5.1%) did not require desmopressin after their first post-operative week.

### Characteristics of patients developing diabetes insipidus

The characteristics of the two groups are shown in Table 2. The DI group were significantly younger (*p* < 0.001), but there was no significant difference in gender distribution between the two groups. There was no significant difference in lesion size between the groups, with the majority of lesions exceeding 1 cm in diameter in both groups. Post-operative medications which may influence AVP levels are given in Table 2.

**Table 2:** Characteristics of patients who developed DI (‘DI’), and those who did not develop DI (‘no DI’)

	No DI (*n* = 67)	DI (*n* = 11)	*p* value
Age	55.9 [22–85]	46.4 [35–69]	<0.001
Gender			
Male	38 (56.7%)	3 (27.3%)	0.070
Female	29 (43.3%)	8 (72.7%)	
Histology			
Gonadotroph	34 (55.2%)	3 (27.3%)	
Corticotroph	4 (6.0%)	2 (18.2%)	
Somatotroph	6 (9.0%)	1 (9.1%)	
Lactotroph	4 (6.0%)	0	
Plurihormonal	6 (9.0%)	2 (18.2%)	
Hormone inactive	2 (3.0%)	0	
Craniopharyngioma	1 (1.5%)	2 (18.2%)	
Rathke’s cyst	6 (9.0%)	0	
Other	4 (6.0%)	1 (9.1%)	
Lesion size > 1 cm	62/67 (92.5%)	10/11 (90.9%)	0.851
Post-operative medications			
Opiate analgesia	58/67 (86.6%)	9/11 (81.8%)	0.675
Anti-hypertensive agents			
ACE inhibitor/ARB	20/67 (29.9%)	4/11 (36.4%)	0.664
Calcium channel blocker	13/67 (19.4%)	2/11 (18.2%)	0.924
Alpha blocker	5/67 (7.5%)	0/11	0.349
Beta blocker	4/67 (6.0%)	0/11	0.405
Clonidine	1/67 (1.5%)	0/11	0.683
Anti-emetics			
Ondansetron	26/67 (38.8%)	6/11 (54.5%)	0.325
Diuretic	6/67 (9.0%)	0/11	0.302
Proton pump inhibitor	16/67 (23.9%)	4/11 (36.4%)	0.380
Psychiatric medication			
SSRI	3/67 (4.5%)	0/11	0.474
SNRI	1/67 (1.5%)	0/11	0.683
Aripiprazole	1/67 (1.5%)	0/11	0.683
Olanzapine	1/67 (1.5%)	0/11	0.683
Haloperidol	1/67 (1.5%)	0/11	0.683
Tricyclic antidepressant	0/67	1/11 (9.1%)	**0.013**

Percentages shown are relative to the total number in that group. Post-operative medications refers to those administered in the first post-operative week

*ACE* angiotensin converting enzyme, *ARB* angiotensin receptor blocker, *SSRI* selective serotonin reuptake inhibitor, *SNRI* serotonin and noradrenaline reuptake inhibitor

Bold values indicates statistically significant values (*p* < 0.05).

### Copeptin results

A total of 44 patients had samples collected at all time points, although two did not have samples at the day 8 and/or 6 weeks. A further 34 patients had samples analysed pre-operatively and at day 1 post-operatively only.

**Fig. 1:**
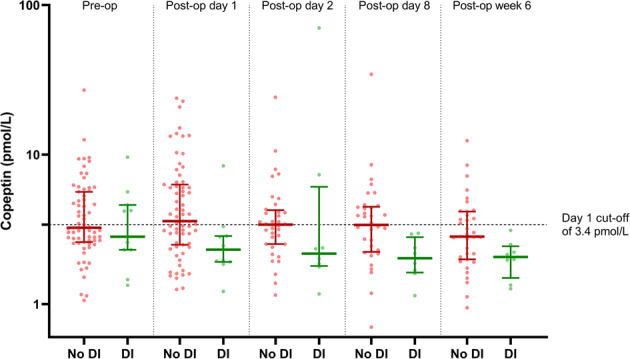
Comparison of copeptin results pre-operatively and at day 1, day 2, day 8 and week 6 between patients who developed DI (DI, in green) and those who did not (no DI, in red). Circles represent individual values, while horizontal lines represent the median values (thick line) and interquartile range (thin lines). A dashed line highlighting the proposed day 1 cut-off value of 3.4 mol/l is shown

Table 3 outlines the copeptin results for the DI versus non-DI group at each time point. As expected, pre-operative copeptin levels did not differ significantly between the two groups. A difference is evident at day 1 post-operatively, with copeptin levels being lower in the DI group, and this is also seen at day 8 and 6 weeks post-operatively. There is no significant difference seen at day 2, largely due to the presence of an outlier result. The copeptin results are displayed graphically in Fig. 1. We observed that, at day 1, a copeptin level of less than 3.4 pmol/l is 91.0% sensitive for detecting DI but only 55.2% specific.

**Table 3:** Copeptin levels are measured time points

Time	Copeptin—no DI	Copeptin—DI	*p* value
Pre-TSA (*n* = 78)	3.24 [*n* = 67] (IQR 2.63–5.46)	2.82 [*n* = 11] (IQR 2.31–4.41)	0.43
Day 1 (*n* = 78)	3.59 [*n* = 67] (IQR 2.52–6.21)	2.31 [*n* = 11] (IQR 1.93–2.84)	**0.01**
Day 2 (*n* = 44)	3.4 [*n* = 36] (IQR 2.52–4.16)	2.17 [*n* = 8] (IQR 1.85–3.61)	0.13
Day 8 (*n* = 42)	3.38 [*n* = 34] (IQR 2.3–4.44)	2.03 [*n* = 8] (IQR 1.67–2.52)	**0.009**
6 weeks (*n* = 43)	2.83 [*n* = 35] (IQR 2.03–4.04)	2.06 [*n* = 8] (IQR 1.81–2.29)	**0.04**

Copeptin values for non-DI and DI groups at the various sampling time points, with associated *p* values. Median values are shown with ranges and interquartile range (IQR) in brackets. All values are in pmol/l

*TSA* trans-sphenoidal adenomectomy

Bold values indicates statistically significant values (*p* < 0.05).

**Fig. 2:**
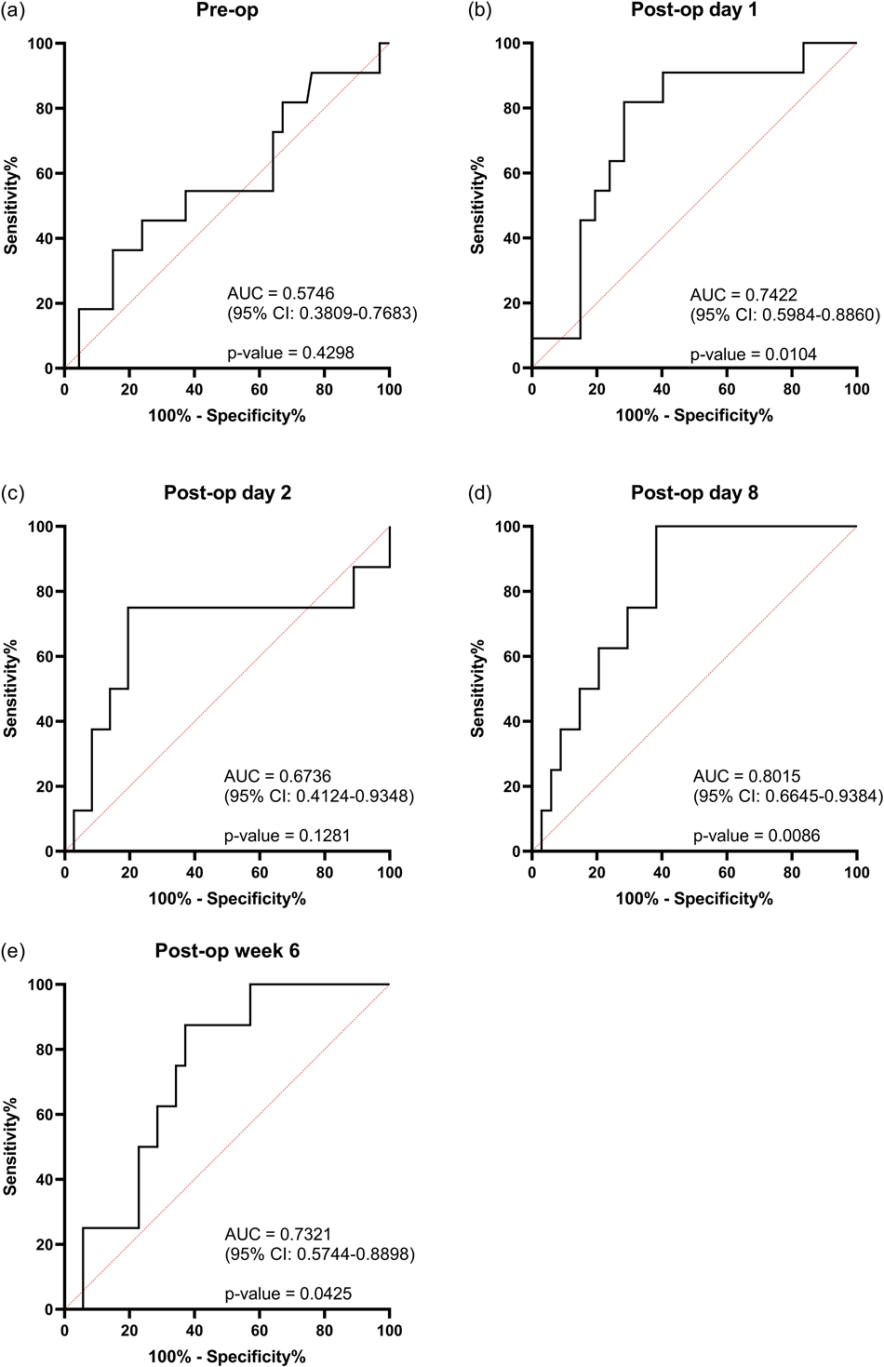
Receiver-operative characteristic (ROC) curves for copeptin pre-operatively (**a**) and at day 1 (**b**), day 2 (**c**), day 8 (**d**) and week 6 (**e**). Curves have been generated using the Wilson/Brown method and are shown in black. Area under curve (AUC) and *p* value also shown

The ROC curves are shown in Fig. 2. The best predictive value is observed at day 1 (AUC = 0.74) and day 8 (AUC = 0.80), while predictive value is also seen at 6 weeks. There is no significant predictive value pre-operatively, while the AUC at day 2 does not differ significantly from equivalence due to the outlier result. Interestingly, there was no significant predictive value of sodium at day 1 (AUC = 0.6811, *p* = 0.0553) and no correlation between sodium and copeptin at this time point (*p* = 0.5003; sodium range 126–148 mmol/l). At this time point, seven patients (9.0%) had hyponatraemia, defined as sodium <135 mmol/l, consisting of two in the DI group (18.1%) and five in the non-DI group (7.5%).

A comparison of the median results in DI and non-DI results at the various time points can be seen in Fig. 1. In patients who do not develop DI, a post-surgical rise in copeptin levels is observed, falling by the 6 week time point. By contrast, the median copeptin level in the DI group falls post-operatively and remains low at subsequent checks. However, analysis of patients with samples collected at all five observation time points using the Friedman test shows no significant difference between time points in both the DI group (*p* = 0.7113, *n* = 9) and non-DI group (*p* = 0.1475, *n* = 33).

**Fig. 3:**
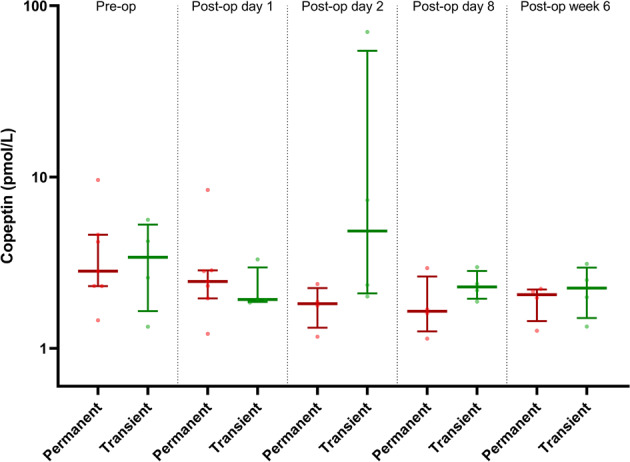
Comparison of copeptin results between those with transient DI (transient, green) and those with permanent DI (permanent, red) at the pre-operatively and at day 1, day 2, day 8 and week 6. Circles represent individual values, while horizontal lines represent the median values (thick line) and interquartile range (thin lines)

When the DI group is separated into permanent versus transient DI, there is a tendency for the permanent DI group to have lower copeptin levels from day 2 onwards (Fig. 3), but the small numbers in the groups mean that no significant difference is demonstrated.

It is notable that copeptin results were higher pre-operatively and at day 1 post-TSS in those with an eGFR <30 ml/min/1.73 m^2^ (pre-operative mean 7.79 pmol/l, day 1 mean 17.95 pmol/l) and heart failure (pre-operative mean 15.14 pmol/l, day 1 mean 13.4 pmol/l). No patients with low eGFR, heart failure or type 2 diabetes developed DI.

## Discussion

We demonstrate that the greatest value of copeptin is in the exclusion of DI following TSS. Copeptin values ≥3.4 pmol/l at day 1 post-operation help to exclude DI, with only 1/38 patients having a value above this threshold going on to develop DI—giving a negative predictive value of 97.4%. The high sensitivity and relatively poor specificity of copeptin is in keeping with other ‘rule-out’ tests, such as the D-dimer test [12, 13]. While this makes copeptin potentially useful in exclusion of DI, the low specificity means copeptin has little value in ‘ruling-in’ or diagnosing DI, in keeping with previous data [9, 14].

We propose that post-pituitary patients with copeptin >3.4 pmol/l at day 1 are candidates for early discharge after surgery—in our cohort 37/67 patients without DI had a day 1 copeptin value of greater than 3.4 pmol/l and may have been discharged 24 h post-operatively if they were otherwise well. This would obviate the need for a further 24 h clinical and biochemical monitoring which has been our usual practice. A prolonged length of stay has been previously reported in a meta-analysis of studies between 1992 and 2013, which showed that the mean length of stay was 3.3 days for patients undergoing endoscopic pituitary surgery and 6.3 days for those undergoing microscopic surgery [14]. Given current demands on a resource-poor publicly funded system, identification of patients who fulfil early discharge criteria enabling shorter inpatient stays is clearly desirable.

In this group of 78 patients undergoing TSS, we find a 14.1% incidence of post-operative DI, which is in line with published data [15]. Although the DI group were significantly younger than the non-DI group, previous studies have not demonstrated a relationship between copeptin levels and age [16]. In our study, there was no significant difference in the gender distribution between DI and no DI groups—baseline copeptin levels have been reported as higher in males, but this has not been shown to be true of stimulated copeptin levels [7]. Given no patients with low eGFR, heart failure or diabetes mellitus developed DI, we were unable to assess whether these conditions alter diagnostic cut-offs. However, the effect of these on copeptin levels in this setting warrants further investigation prior to use in these groups.

This study has some limitations. Firstly, our study used retrospective batch analysis of samples collected at routine time points as opposed to real-time analyses/assay. To our knowledge, no centres are currently using the copeptin assay in real-time, with most freeze-thawing samples for later batch analysis. In order to evaluate if contemporaneous copeptin results will affect day-to-day management and discharge of pituitary surgical patients, the assay will need to be used in real-time and our centre has now set up such a facility to provide results on day 1 post-TSS.

Secondly, we may have missed the ‘peak’ copeptin provoked by manipulation of the gland. Berton et al. have shown that 1 h post-extubation samples have a greater predictive value for diagnosis of DI [17]. In support of this, the Christ-Crain group demonstrated greatest discriminative power in samples taken at less than 12 h following operation [10], although recent work by Vanasuntorn et al. has shown good predictive value within the first 24 h after surgery [18]. We are keen to limit blood sampling for patients but it may be that an earlier sampling time, soon after surgery, would yield a more discriminative copeptin threshold for discharge.

A further consideration is the use of post-operative fluid restriction. The rationale for this is that it prevents masking of the diagnostic biochemical features of DI through excessive water intake. This protocol necessitates close clinical and biochemical monitoring, to prevent dehydration, with restriction lifted if patients show any features of DI.

Finally, it is worth noting that the small numbers used preclude use of copeptin as the sole tool for determining discharge at day 1. It should be used in conjunction with other clinical and biochemical parameters to make an overall assessment until further evidence on use of copeptin is available.

## Conclusion

We confirm that post-pituitary surgery copeptin measurement is useful to exclude DI early post-operatively and propose it as a potential tool to identify patients suitable for early discharge. It will be important to conduct future work on earlier sampling times, with real-time assay, and combine these results with other clinical and biochemical parameters to provide a robust dataset at 24 h post-pituitary surgery enabling selection of patients for safe early discharge.
